# The Canadian birth place study: examining maternity care provider attitudes and interprofessional conflict around planned home birth

**DOI:** 10.1186/1471-2393-14-353

**Published:** 2014-10-28

**Authors:** Saraswathi Vedam, Kathrin Stoll, Laura Schummers, Nichole Fairbrother, Michael C Klein, Dana Thordarson, Jude Kornelsen, Shafik Dharamsi, Judy Rogers, Robert Liston, Janusz Kaczorowski

**Affiliations:** Faculty of Medicine, University of British Columbia, B54-2194 Health Sciences Mall Vancouver, Vancouver, BC V6T 1Z3 Canada; Department of Epidemiology, Harvard School of Public Health, 677 Huntington Avenue, Boston, MA 02115 USA; Island Medical Program, University of British Columbia, Royal Jubilee Hospital, Room 141 Eric Martin Pavilion, 2328 Trent Street, Victoria, BC V8R 4Z3 Canada; Child and Family Research Institute, 950 West 28th Avenue, Vancouver, BC V5Z 4H4 Canada; Centre for Rural Health Research, University of British Columbia, Suite 300 David Strangway Bldg, 5950 University Boulevard, Vancouver, BC V6T 1Z3 Canada; Department of Family Practice, University of British Columbia, Suite 300 David Strangway Bldg, 5950 University Boulevard, Vancouver, BC V6T 1Z3 Canada; Midwifery Education Program, Ryerson University, 99 Gerrard Street East Room SHE-582, Toronto, ON M5B 2L4 Canada; Faculty of Medicine, University of British Columbia, C420 - 4500 Oak Street, Vancouver, BC V6H 3N1 Canada; Department of Family and Emergency Medicine, University of Montreal Hospital Research Centre (CRCHUM), Tour Saint-Antoine, Office: S03.416, 850 rue St-Denis, Montreal, QC H2X 0A9 Canada

**Keywords:** Home childbirth, Birth place, Inter-professional collaboration, Scale development, Physicians, Midwives, Psychometrics

## Abstract

**Background:**

Available birth settings have diversified in Canada since the integration of regulated midwifery. Midwives are required to offer eligible women choice of birth place; and 25-30% of midwifery clients plan home births. Canadian provincial health ministries have instituted reimbursement schema and regulatory guidelines to ensure access to midwives in all settings. Evidence from well-designed Canadian cohort studies demonstrate the safety and efficacy of midwife-attended home birth. However, national rates of planned home birth remain low, and many maternity providers do not support choice of birth place.

**Methods:**

In this national, mixed-methods study, our team administered a cross-sectional survey, and developed a 17 item *Provider Attitudes to Planned Home Birth Scale (PAPHB-m)* to assess attitudes towards home birth among maternity providers. We entered care provider type into a linear regression model, with the PAPHB-*m* score as the outcome variable. Using Students’ *t* tests and ANOVA for categorical variables and correlational analysis (Pearson’s r) for continuous variables, we conducted provider-specific bivariate analyses of all socio-demographic, education, and practice variables (n=90) that were in both the midwife and physician surveys.

**Results:**

Median favourability scores on the PAPHB*–m* scale were very low among obstetricians (33.0), moderately low for family physicians (38.0) and very high for midwives (80.0), and 84% of the variance in attitudes could be accounted for by care provider type. Amount of exposure to planned home birth during midwifery or medical education and practice was significantly associated with favourability scores. Concerns about perinatal loss and lawsuits, discomfort with inter-professional consultations, and preference for the familiarity of the hospital correlated with less favourable attitudes to home birth. Among all providers, favourability scores were linked to beliefs about the evidence on safety of home birth, and confidence in their own ability to manage obstetric emergencies at a home birth.

**Conclusions:**

Increasing the knowledge base among all maternity providers about planned home birth may increase favourability. Key learning competencies include criteria for birth site selection, management of obstetric emergencies at planned home births, critical appraisal of literature on safety of home birth, and inter-professional communication and collaboration when women are transferred from home to hospital.

## Introduction

The current unprecedented rates of operative delivery and intrapartum interventions have led to a global call for research and reflective practice focused on improving rates of physiologic labour and birth [1–4]. These evidence-based position statements advocate for birth environments and models of maternity care that prioritize the judicious use of obstetric technology when caring for healthy women and newborns. Women’s preference for planned home and birth center births is related to their ability to realize optimal maternal and newborn outcomes while minimizing utilization of interventions [5–8]. Interest in births at home and in birth centers is on the rise in high resource countries [9–11]. Recent investigations [9, 12–17] report that optimal outcomes and reduced interventions at planned home births lead to significant cost-savings when care is fully integrated into the health care system [18, 19]. As a result, health policy leaders and regulators have supported increased access to high quality maternity care at planned home births [20].

Although professional bodies may call for expansion of settings that promote normal, physiologic birth, their constituents, maternity care providers, do not share a unified approach to management of labor and birth. Previous research in Canada has demonstrated significant polarity in attitudes towards common maternity care practices, with obstetricians and family physicians favouring hospital deliveries and obstetric interventions, and midwives favoring a low-intervention approach to labor and birth, either at home or the hospital [21]. Such divergence in attitudes can contribute to perceptions of interprofessional conflict among childbearing women [22] and thus reduce the culture of safety for women and families who choose planned home birth [17, 23].

To examine the sources of potential conflict among maternity providers around women’s choice of birth setting, we designed the Canadian Birth Place Study, a mixed methods evaluation of systemic factors, as well as personal, educational and practice experiences, that are associated with attitudes towards home birth among family physicians, obstetricians, and midwives [24, 25]. In this paper we describe the development of a scale that measures attitudes of multidisciplinary care providers towards home birth, subsequent psychometric evaluation of that scale and bivariate and multivariate analysis of factors that are associated with attitudes towards homebirth.

## Background

### The environment for practice

Currently, in most provinces across Canada, pregnant women can choose a family physician, obstetrician or a midwife as a primary maternity provider, and their comprehensive pregnancy and birth care is funded by provincial insurers. In some jurisdictions obstetricians, who previously provided only consultant services, are also providing primary maternity care; but at present there is a shortage of any type of physicians in active maternity practice in both urban and rural settings [26]. All obstetricians, and those family physicians who provide intrapartum care, deliver exclusively in hospitals. Some provinces and institutions have instituted regulatory sanctions which prohibit physicians from attending home births.

Since licensure became available in 1993, registered midwives have increased their presence in 8 of 10 provinces and 1 of 3 territories, but the demand for their services exceeds availability. Midwives offer choice of birth place and they are required, by their regulatory bodies, to attend births in all available settings: home, hospital, birth centre. Canadian national policy has embraced midwifery and choice of birth place by instituting reimbursement schema and regulatory guidelines to ensure access to midwives in all settings. However, in some provinces, midwives have been incorporated into the health care system only recently. In Quebec, midwives attend births almost exclusively in birth centres.

National rates of planned home birth are less than 2 percent, and there is significant variation across jurisdictions in access to care, consultation, and collaboration across birth settings [27]. A majority of Canadian women begin antenatal care with family physicians who do not offer intrapartum care, and so must seek another provider mid-pregnancy. Also, because of maternity care provider shortages, women sometimes must place themselves on waiting lists until and if spaces become available. Hence, several types of providers can be involved in the women’s decision making around birth place [28, 29], and those who desire a planned home birth may not be able to access a registered midwife.

Exposure to various birth settings within undergraduate and postgraduate education programs differs among the health professional programs. In medical schools, only hospital-based intrapartum clinical practicums are incorporated into the required core curricula, whereas birth centre and home birth practicums are rarely offered and never required. In contrast, attendance at home birth is a standard requirement in registered midwife (RM) education programs. To date, in Canada, birth centres are integrated into the system of care only in the province of Quebec, and only midwife learners have routine access to clinical education experiences in those centres.

The Canadian Birth Place study was designed to evaluate the opinions and experiences with planned home births among all types of maternity providers, and the potential impact of these attitudes on interprofessional collaboration across birth settings. Few previous studies have explored attitudes towards home birth, and none used a quantitative instrument with validated psychometric properties to measure attitudes and their covariates across the maternity professions [30–33]. In 2007, Vedam et al. developed and evaluated a 20 item *Provider Attitudes towards Planned Home Birth scale* (*PAPHB*) [34], and applied it to Certified Nurse-Midwives (CNMs), in the United States [35]. Educational and practice exposure to planned home birth, age, inter-professional relationships, and financial concerns emerged as significant covariates of attitudes. CNMs’ lack of confidence in their own ability to manage complications in the home was associated with less favourable attitudes and less willingness to provide care in the home setting. This previous research informed the survey and scale development for the Canadian Birth Place Study.

## Methods

### Study design

We assessed the psychometric properties of a comprehensive survey of attitudes towards planned home birth among physicians and midwives. The survey items were adapted to the Canadian context and for all maternity provider groups from an instrument which was developed and validated with a single provider group (midwives) in the US [35]. The survey was designed to measure the construct “attitudes to home birth” in four subdomains: the safety of planned home birth, maternal/newborn outcomes from planned home births, maternal/newborn benefits from home births, and inter-professional experiences and engagement with home birth practice. Content validation of the Canadian Birth Place survey instrument by provider-specific expert panels, and pilot testing by physician and midwife resident learners, are described elsewhere [24]. The study was funded by the Canadian Institutes of Health Research and led by a multi-disciplinary team of investigators. Ethics approval was obtained from the University of British Columbia.

In 2010 (August-October), during the quantitative phase of the Canadian Birth Place study, we invited all Canadian obstetricians (n=835), registered midwives (n=759), and a random sample of family physicians (n=3000) to complete this comprehensive survey that included information about their demographic profile, education and practice experiences with home birth (39 items), and 48 attitude items. Response options for each attitude item ranged from 1 (strongly disagree) to 5 (strongly agree). We obtained direct mail and fax contact information for all registered midwives in Canada via provincial regulatory college rosters, and all obstetricians who provided intrapartum care via the Society for Obstetricians of Gynaecologists of Canada (SOGC). A random and geographically stratified sample of family physicians (10% of total) was generated, using a national physician directory. Potential respondents were invited to participate in the survey, via multiple avenues (e-mail, direct postcards, posters, and fax), and reminders (at 2–3 week intervals) [24]. The Canadian Association of Midwives and SOGC sent an email link to the survey to their memberships, on behalf of the researchers. We adhered to STROBE guidelines during the research and manuscript development process.

### Scale construction

Results for each provider type were analyzed to select items for an attitude scale that could be used as an outcome measure. We first examined the correlation between each item score and the total score of the 48 attitude items (corrected item-total correlation); this was done for each care provider group separately. The 17 items that had Pearson correlational coefficients exceeding 0.3 for each care provider group (obstetricians, midwives, family physicians) were retained for inclusion in the scale. Next, we calculated internal consistency for the 17 items. The overall scale alpha was excellent for the full sample (Cronbach’s alpha = 0.974), and good for each care provider group (MW alpha = 0.795; GP alpha = 0.922; OB alpha = 0.839). In an unweighted least squares factor analysis, the screeplot suggested a one factor solution for each care provider group, as well as the overall sample. This meant that together the 17 items measure a single construct and should not be split into subscales. Factor loadings for the 17 items ranged from 0.326 to 0.759 for obstetricians, to 0.347 to 0.832 for GPs, and 0.269 to 0.628 for midwives. Item to total correlations and factor loadings for the full sample are reported in Table 1. The scale was named ‘*Provider Attitudes to Planned Home Birth Scale – m’* (for multidisciplinary). Higher scores indicate more favourable attitudes towards planned home birth.

**Table 1:** **Provider attitudes towards planned home birth - M scale items: item to total correlations and factor loadings (n=825)**

		Corrected item to total correlations	Factor loadings
1	Registered Midwives have sufficient skills to handle most emergencies safely at planned home births.	0.881	0.405
2	Women who give birth in the hospital are more likely to experience morbidity associated with medical interventions than women who give birth at home.	0.672	0.459
3	First time mothers should have the option of having a planned home birth.	0.896	0.555
4	I would feel comfortable if a close family member planned to give birth at home.	0.933	0.486
5	I am more comfortable with hospital birth than I am with planned home birth (reverse scored).	0.862	0.455
6	It worries me when people I care about decide to have planned home births (reverse scored).	0.873	0.499
7	There is scientific evidence that supports the greater safety of hospital births compared to planned home births (reverse scored).	0.809	0.538
8	Women who plan home births tend to be risk takers (reverse scored).	0.716	0.404
9	Planned home birth is not as safe as hospital birth (reverse scored).	0.901	0.628
10	Because of the risk of postpartum hemorrhage, the home is not an ideal birth setting (reverse scored).	0.897	0.477
11	I would consider having my own (or my partner’s) planned home birth with a Registered Midwife.	0.912	0.417
12	I am more comfortable providing intrapartum care in the hospital because of the personnel and equipment available only in the hospital (reverse scored).	0.883	0.510
13	A move towards more planned home births in this country would save our health care system a significant amount of money.	0.773	0.407
14	Even in urban areas, planned home births are less safe because of the amount of time it takes to transfer mothers/babies to hospital (reverse scored).	0.875	0.530
15	A woman who plans a hospital birth is more likely to have an unnecessary cesarean section than a woman who plans a home birth.	0.580	0.392
16	A mother’s cultural background is easier to respect at home births than hospital births.	0.467	0.269
17	I like attending planned home births.	0.889	0.482

### Data analysis

Care provider type was dummy coded with midwives as the reference category. The care provider variable was then entered into a linear regression model, with the 17 item PAPHB-*m* scale score as the outcome variable, to determine how much variance in attitudes towards planned home birth could be accounted for by type of care provider alone. We also conducted care provider-specific bivariate analyses of all socio-demographic, education, and practice variables that were included in both the midwife and physician surveys (n=90). We used Students’ *t* test for categorical variables with two levels, one way ANOVA for categorical variables with more than 2 levels, and correlational analysis (Pearson’s r) for continuous variables. These parametric tests are robust to violations of the normality assumption, as long as the variables are independent [36, 37].

After 17 items were summed to form the PAPHB-*m* attitude scale, 31 Likert items remained. All survey items including the remaining Likert items had been subjected to rigorous content validation by maternity care experts and deemed important to the measurement of home birth attitudes [24]. Although these items were not included in the scale, many assessed important dimensions of attitudes towards home birth, such as inter-professional practice and relationships, perceptions of the safety of home birth, educational exposure to planned home birth and liability concerns. To assess the relative importance and association of these factors with the PAPHB-*m* scale score, we excluded factors that would differ according to location (such as regulatory status and availability of emergency services), and calculated Pearson’s r (correlational coefficient) for the remaining 18 items across care provider groups**.** A negative coefficient indicates that the item is correlated with more unfavourable attitudes; a positive coefficient means that the item is associated with more favourable attitudes towards home birth (see Table 2).

**Table 2:** **Associations of the 17 item scale score with selected attitude items, reported by care provider type (n=825)**

	MWs		GPs		OBs	
	r value	p value	r value	p value	r value	p value
I prefer the familiar physical setup of the hospital to the unknown and varied discovery rate, we accepted findings with physical conditions of individual homes.	-.507	<0.001	-.518	<0.001	-.173	.008
Liability concerns reduce my willingness to attend PHBs.	-.333	<0.001	-.259	.002	-.028	.672
The home setting is an ideal birth environment for mother-baby bonding.	.304	<0.001	.508	<0.001	.510	<0.001
Resuscitation of the term newborn is as effective in the home setting as in the hospital setting.	.297	<0.001	.367	<0.001	.427	<0.001
It is easier to maintain individualized care at a PHB than at a planned hospital birth.	.295	<0.001	.448	<0.001	.470	<0.001
Women who have PHBs have a greater risk of perinatal loss than women who have planned hospital births.	-.263	<0.001	-.645	<0.001	-.451	<0.001
Birth can only be described as normal retrospectively.	-.242	<0.001	-.447	<0.001	-.265	<0.001
PHB is more empowering for the mother than hospital birth.	.233	<0.001	.430	<0.001	.398	<0.001
There are more effective pain management options for birth in the hospital.	-.230	<0.0001	-.235	.005	-.291	<0.001
My midwifery/medical school faculty/mentors were positive when discussing PHB.	.189	<0.0001	-.032	.712	.159	.015
Providers who attend HBs in their practice are at a higher risk of lawsuits than those who only attend hospital births.	-.186	<0.0001	-.361	.000	-.340	.000
HB clinical experiences within educational programs are only important for those providers who work in HB settings.	-.177	<0.0001	-.240	.004	-.098	.134
There are evidence-based criteria that can help providers to identify women who are good candidates for HB.	.172	<0.0001	.549	.000	.488	.000
HB providers experience disapproval from hospital-only maternity care providers.	.142	.002	-.014	.867	.073	.265
Physicians [midwives] have sufficient skills to handle most emergencies safely at PHBs.	-.063	.180	.445	<0.001	.304	<0.001
Physicians who attend PHBs are risking formal censure.	-.060	.203	-.168	.048	-.223	.001
There are physicians in my area who are comfortable providing consultation/accepting transfers from RMs attending PHBs.	.056	.234	.249	.003	.156	.016
When I provide consultation to midwives/[When I consult with a physician] for intrapartum clients transferring from PHB, I feel uncomfortable.	-.015	.745	-.411	<0.001	-.257	<0.001

Because of the large number of analyses we conducted and the associated risk of an elevated false discovery rate, we accepted findings with a p value =/<0.001 as significant. Nonetheless, we discuss findings that had a p value of < 0.05 as trends.

## Results

We received responses from 950 care providers. We excluded gynecologists who had never engaged in intrapartum care, as well as test submissions and incomplete surveys. The final sample size was comprised of 825 care providers: 451 midwives, 235 obstetricians and 139 family physicians. The response rate was 18.1% (of invited care providers). Response rates were highest for midwives (59.4%) and lowest for family physicians (4.6%). Midwives were the youngest group of providers (mean age = 41.3) followed by obstetricians (mean age = 46.5) and family physicians (mean age = 48.1). Most midwives who responded to the survey were female (99.3%), compared to 55.6% of obstetricians and 54.0% of family physicians. Physicians had very little exposure to home birth during medical school and practice; e.g. 2.2% of family physicians and < 1% of obstetricians learned about planned home birth as part of their core curriculum, whereas 82.7% of midwives reported that their curriculum included coursework about planned home birth. While 99.1% of midwives reported providing intrapartum care in the home, only 5% of family physicians and < 1% of obstetricians had this experience.

Socio-demographic factors that were associated with more favourable attitudes towards home birth among obstetricians included: being female (35.6 versus 33.0, *t* = 2.08, *df* = 232, *p* = 0.04), having obtained a graduate degree (Master’s or PhD) (38.4 versus 33.2, *t* = -3.52, *df* = 233, *p* = 0.001) and reporting involvement in research (36.4 versus 33.6, *t* = -2.04, *df* = 233, *p* = 0.04).

### Scale scores by care provider group

The Provider Attitudes to Planned Home Birth Scale *– m* has a hypothetical range of 17–85, with scores below 51 indicating unfavourable attitudes towards home birth and scores exceeding 68 indicating favourable attitudes. Scores between 51 and 67 represent neutral attitudes. These categories were derived as follows: A score of 3 on an individual item indicated a neutral attitude towards that item, scores of 1 and 2 denoted disagreement with the item and scores of 4 and 5 indicated agreement. By multiplying these response options by the number of scale items we created cut off scores that allowed us to better interpret where each care provider group falls on the scale.

Midwives (*n* = 451) scored an average of 78.65, with less dispersion around the mean (SD = 6.28) compared to the other two care provider groups The median score of midwives was 80 (5th, 95th percentiles: 68, 85). Almost all midwives fell into the favourable range of the scale.

Obstetricians (*n*=239) had a mean score of 34.43 on the scale (SD = 9.75) and a median score of 33 (5th, 95th percentiles: 21, 51). Most obstetricians fell into the unfavourable range; obstetricians who scored in the 95th percentile on the scale held neutral attitudes towards home birth, on average.

Family physicians (*n*=139) scored an average of 41.58 points with the most dispersion around the mean (SD = 14.25). The median score of family physicians was 38 (5th, 95th percentiles: 22, 71). Family physicians, on average, scored in the unfavourable range; however the 95th percentile of family physicians scored in the favourable range.

**Figure 1:**
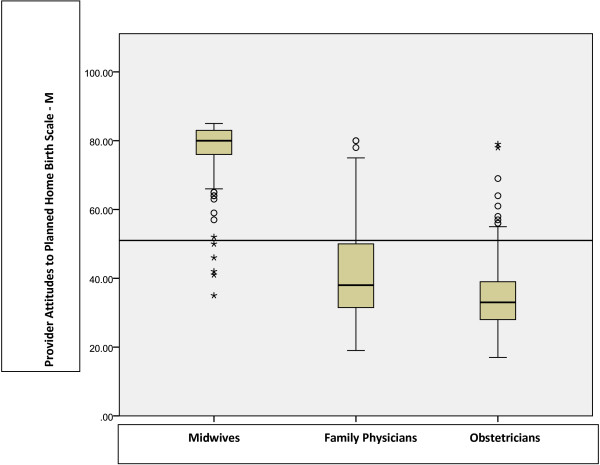
**Favourability towards planned home birth: Median and interquartile range of PAPHB-** ***m*** **scores by care provider group (n=825).** The horizontal line inside each box represents the median score for each provider group, and the upper and lower boundaries of each box represent the upper and lower quartiles. The vertical lines represent the range of scores, excluding outliers, which are represented by open circles and asterisks.

The box plot (Figure 1) displays the median attitude scores by care provider group. The majority of midwives (98.9%) scored above neutral, as well as 25.2% of family physicians and 5.5 % of obstetricians. Care provider type accounted for 84.2% (adjusted R^2^) of the variance in attitudes towards planned home birth. Family physicians and obstetricians were significantly less favourable towards planned home birth compared to midwives (p < 0.001). The large proportion of variance accounted for by care provider type led to the decision to examine covariates of attitudes separately for each provider group.

### Exposure to home birth

Internationally educated midwives were significantly less favourable towards planned home birth than midwives who completed their education in Canada (77.3 versus 79.2, *t* = 2.55, *df* = 449, *p* = 0.003). All midwives reported exposure to home birth through clinical practice. The 2 obstetricians who had been present at one or more home deliveries in a support role during practice had significantly higher scores compared to obstetricians without this experience (67.0 versus 34.1; t = - 4.98; p < 0.001). Family physicians who had attended at least one planned home birth as an observer or support person during clinical practice had more favourable attitudes towards homebirth (68.0 versus 41.2; t = - 2.70, p = 0.008).

Family physicians who graduated from medical school after the introduction of registered midwives in Ontario (1993), displayed more favourable attitudes towards planned home birth compared to physicians who graduated prior to 1993 (45.5 versus 39.4, *t* = -2.44, *df* = 137, *p* = 0.02). Family physicians who were taught by planned home birth providers during medical school were more favourable towards planned home birth compared to physicians without this educational exposure (48.7 versus 40.7, *t* = 2.06, *df* = 137, *p* = 0.04). The single family physician who attended one or more home deliveries in a support role during his/her education had a scale score of 80.0 compared to physicians who had not attended home deliveries in this role (41.3).

### Factors most correlated with scale scores by provider type

Factors that were most strongly correlated with scale scores (Table 2) were similar among the three groups of providers, but there were some notable differences between midwives and all physicians. Among physicians agreement that “there are evidence based criteria to help providers identify women who are good candidates for home birth”, and agreement that “physicians have sufficient skills to handle most obstetric emergencies safely at home” were correlated very strongly with higher scale scores. Higher scales scores were also correlated with physicians who did *not* believe resuscitation was more effective in the hospital. These factors were also significantly correlated among midwives but not as strongly.

Among both physicians and midwives, favourability to home birth was also positively correlated with the beliefs that mother-infant bonding was easier in the home, that home birth is more empowering, that it is easier to individualize care in the home, and that resuscitation of the newborn was *not* more effective in the hospital. However, correlations with favourability were strongest among physicians.

Lower scale scores amongst midwives were correlated most strongly with their stated preference for the familiar physical set up of the hospital. Beliefs that home birth leads to higher risks of perinatal loss and lawsuits correlated with lower scale scores but these correlations were stronger for physicians than midwives. Lower scores for all types of providers were correlated with those who agreed that “there are more effective pain management options in the hospital” and “birth can only be described as normal in retrospect”.

### Inter-professional practice

Bivariate analyses elicited some regional variations in factors associated with scale scores. The 8 obstetricians who had ever shared intrapartum care with a registered midwife in the home setting had significantly higher scores on the scale (43.3 versus 34.1, *t* = -2.637, *p* = 0.009). There was a trend towards lower scores among physicians who practiced in areas of the country where planned home birth is prohibited (obstetricians: 31.3 versus 35.4, *t* = -2.006, *p* = 0.046; family physicians: 35.2 versus 43.4, *t* = -2.213, *p* = 0.03).

Midwives who work in communities where obstetricians practice had more favourable views about planned home birth compared to midwives who practice in communities without obstetricians (78.9 versus 76.4, *t* = - 2.60, *df* = 449, *p* = 0.01). Similarly, midwives who practice in communities with a provincial referral centre (i.e. highest level of care) were significantly more favourable towards home birth (79.7 versus 77.8, t = -3.20, df = 449, p = 0.01). Midwives who had experience practicing in birthing centers also had higher scale scores (79.7 versus 78.0; t = - 2.94, p = 0.004).

Physicians who reported feeling uncomfortable during consultations with midwives (for intrapartum clients transferring from a planned home birth) were significantly less favourable towards home birth. Similarly, physicians who practice in communities where some physicians are comfortable providing consultations and/or accepting transfers from RMs attending home births had significantly higher scores on the scale (see Table 2).

## Discussion

In this Canadian Birth Place study, midwives had the most favourable scores on the home birth attitudes scale, with the least amount of variation in attitudes. Physicians were mostly unfavourable, although some family physicians exhibited positive attitudes towards home birth. Very few obstetricians scored above neutral; the ones who did can be considered outliers (see Figure 1).

### Socio-demographic factors

Canadian-trained midwives were more favourable suggesting that the unique aspects of learning within the existing model of care and midwifery education, including requirements for attendance at home birth, had a positive effect. Among obstetricians, female gender, graduate education, and research involvement were significantly associated with higher scores on the scale. This is consistent with Klein’s findings that maternal-fetal medicine specialists and female physicians had more comfort with low intervention approaches [21]. However, since as a group obstetrician scores fell into the unfavourable range, it appears that the overall lack of exposure, and concerns about safety override the effects of demographics and level of education on attitudes towards planned home birth.

### Perceptions of the safety of home birth

Provider beliefs about the normalcy of birth, the evidence basis for selection criteria, the ability to provide skilled emergency care, and risks of loss or liability from home birth were completely aligned with the variance in attitudes by provider type. While more favourability among all providers was correlated with the importance of enhanced maternal-newborn benefits of individualized care, and an environment which facilitates bonding and empowerment, higher scores were aligned with beliefs about the safety of home birth only among midwives.

The effects of education on professional acculturation are apparent in this study. The finding that graduate education affected favourability among obstetricians may mean that fluency with critical appraisal of research methodology, and exposure to emerging evidence may change interpretation of relative risk across birth settings. In their critical analysis of the social and cultural shaping of medical evidence, DeVries & Lemmens assert that, in maternity care, science follows practice, rather than practice being led by emerging science [38]. To illustrate how culture influences the interpretation and uptake of medical evidence, the authors outline historical and cultural factors that have contributed to the acceptance and high rates of planned home birth in the Netherlands. The home birth literature displays significant variations in the quality of study methodologies [39], and the direction of interpretation (safe/unsafe) and publication of results in journals appears to be consistently aligned with type of profession [40]. This conclusion is supported by Hall, Tomkinson & Klein who, through interviews with 56 Canadian maternity care providers in 2008 and 2009, found that care providers present scientific evidence in ways that optimizes women’s compliance with their recommendations, even in non-emergency situations [41]. These findings support the notion that scientific evidence may be understood through a pre-existing lens that accords with different types of care providers’ philosophy of practice and comfort level.

### Exposure

Opportunities to attend more planned home birth in support or observer roles (during education and/or practice) were linked to more favourable attitudes towards planned home birth for physicians. For all types of providers, and especially physicians, more exposure to planned home birth through clinical practice, or in support or observer roles, was associated with more favourable attitudes towards planned home birth. Less favourable attitudes were linked to physicians who practice in jurisdictions where home birth is prohibited. There was a trend towards less negative attitudes towards planned home birth among family physicians who graduated after the introduction of regulated midwifery in Ontario; and Ontario has the longest history of regulated midwifery. This suggests that attitudes towards planned home birth may be linked to exposure to midwifery practice. As midwifery becomes more established in other Canadian provinces and territories, home birth might become more acceptable among physicians.

While divergences in overall attitudes between all physicians and midwives may be partially explained by caseload competition, these differences are more likely a result of clear differences between midwives and physicians with respect to perceptions around safety, confidence in their own ability to provide comprehensive care in the home, and/or differential values regarding physiologic, low intervention approaches to labour and birth management [21, 42]. Opportunities for cross-disciplinary clinical mentorship for primary maternity care providers may translate into increased acceptance of all models of care and may contribute to more collaborative maternity care practice.

### Inter-professional relationships

Notably, almost none of the categorical covariates we examined were associated with neutral or favourable attitudes towards planned home birth among obstetricians, indicating pervasively negative views towards home birth in this group. Even those obstetricians who practice alongside midwives in the same clinic were, at best, unfavourable (average score 37.9), suggesting that their exposure to providers who attend planned home births may not override the influences of their professional culture, education or their own exposure to various sites for birth. The discomfort all provider groups expressed when consulting with each other around home birth cases implies an awareness of differences in their respective philosophies, roles, and approach to practice [25].

While midwives have become accepted members of maternity care teams, home birth remains a contentious issue that can cause friction among providers [25, 43]. Inter-professional communication and collaboration is especially important during urgent transfers from home to hospital. Researchers have found that effective teamwork and communication during critical obstetric events results in fewer intrapartum neonatal and maternal deaths [44–46]. Where such integrated systems are in place in Canada [16], perinatal results are excellent. Lack of role clarity and poor communication are primary determinants of *preventable adverse neonatal and maternal outcomes, including death* [44] *.*

Maternity providers’ attitudes towards each other and towards clinical practices and approaches to care (e.g. technological vs. low-intervention, place of birth) dictate the options available to childbearing families. Misalignments in beliefs about home birth contribute to the theory-practice gap between best available evidence and application to practice. Where collaboration may not be the norm, practitioners may be operating under incorrect assumptions. When physician attitudes are biased against midwifery and home birth and midwives attitudes are in conflict with their consultants, the quality of inter-professional interactions ultimately affects both patient choice and quality of care across birth sites [12, 47].

Health human resource investigations have suggested that inter-professional education and team development can address the weaknesses of the current system. Currently, Midwifery, Nursing, and Medical student cohorts are taught separately how to provide maternity care and graduate without a full understanding of each other’s knowledge base and scopes of practice. However, competencies for interprofessional practice are now recognized as core to preparation for practice in a collaborative team environment [48, 49]. Clinical placements with preceptors who offer home and/or birth centre births, and opportunities for trans-professional elective experiences may increase the favourability of obstetricians and family physicians to the option of planned home birth with midwife attendants, and midwives’ understanding of the constraints that limit physicians’ practice.

### Implications for health systems planning

In Canada, the barriers to multidisciplinary maternity care include differences in provider models and mandates, most acutely noted through disparate remuneration schemes, scopes of practice, and differences in curricula. Curricula for physicians and midwives differ significantly because of differences in the scopes and sites of practice of these health professionals. Less favourable attitudes among both physicians and internationally educated midwives may indicate a need for additional education and/or resources to assist them to become comfortable with the Canadian model of midwifery care, including requirements for home birth practice.

Midwives are required by regulation to provide care in all settings, but they are not available in all communities. In contrast, physicians are restricted to hospital birth by many professional policies and home birth is not easily integrated into office practice. In many jurisdictions significant shortages of human health resources for maternity care may also have an impact on attitudes. Gaps in equitable reimbursement for identical services by different types of professionals may also raise obstacles to effective collaboration when providing maternity care across all settings [50]. All of these factors may contribute to differences in attitudes and philosophy of care.

These divergences have important implications for evolving models of health human resource allocation. In North America, home birth services are associated with higher rates of physiologic birth, reduced use of costly obstetric interventions, and optimal maternal and newborn outcomes [12, 15, 16]. In response to public demand and emerging evidence, both location of birth and types of providers are diversifying [51, 52]. In provinces where midwifery is regulated, 25-30% of midwifery clients plan home births. Rates of planned home birth are somewhat higher among women who reside in rural areas of the province.

Several national initiatives have focused on improving inter-professional collaboration [53, 54]. In 2009, the College of Physicians and Surgeons of BC reversed its previous stance, prohibiting physicians from attending home birth and published new Guidelines on Planned Home Birth which state*, “When a woman is considering planned home birth, physicians play an important role in providing advice and information so that it is an informed choice, considering all the benefits and potential adverse outcomes …Physicians involved in planned home births need to ensure that they have appropriate knowledge, training, equipment and understanding of the assessments necessary in planned home delivery.”*

The development of systems that can deliver high-quality, cost-effective, and equitable maternity care to all populations, must consider both roles and interactions among different types of health professionals, even when women have access to highly-resourced tertiary care centers [55–59].

### Limitations

The sample size for family physicians was low; for this reason findings cannot be generalized to all Canadian family physicians, especially since survey respondents might have more polarized attitudes towards home birth than non-responders. The more rigorous p value of 0.001 was chosen to minimize false discovery due to multiple comparisons. However, the family physician sample was not adequately powered to detect small differences in mean scale scores among family physicians with different socio-demographic attributes and educational and practice experiences around home birth. For this reason, the study should be replicated with a larger sample of physicians. A larger sample would also allow for analysis of regional differences for conditions for practice.

## Conclusions

Even when physicians and midwives practice in different settings, and the course of pregnancy is normal, the nature of perinatal care requires effective communication among all types of maternity providers [53]. The significant differences in attitudes to planned home birth and the covariates of these attitudes suggest that an increased emphasis in health professional education on preparation for practice in all birth places is important for family physicians, and obstetricians, as well as midwives. The data suggest that developing inter-professional competencies around best practice care and communication when collaborating to care for women across several birth settings, may increase favourability among physicians and midwives. Ultimately, understanding the perceptions and actual obstacles to inter-professional practice may inform the design of effective evidence-based models for delivery of maternity care across birth sites.

## Authors’ information

**SV,** RM FACNM MSN Sci D(hc)

Associate Professor, UBC Midwifery

Faculty of Medicine, University of British Columbia

**KS**, PhD

Postdoctoral Fellow, UBC Midwifery

Faculty of Medicine, University of British Columbia

**LS**, SM, SD cand.

Department of Epidemiology, Harvard School of Public Health

**NF**, PhD, RPsych

Assistant Professor, Department of Psychiatry

Island Medical Program, University of British Columbia

**MK**, MD, CCFP, FAAP

Professor Emeritus, Department of Family Practice

Faculty of Medicine, University of British Columbia

**DT**, PhD, RPsych

Lecturer, UBC Midwifery

Faculty of Medicine, University of British Columbia

**JK,** PhD

Co-Director, Centre for Rural Health Research

Assistant Professor, Dept of Family Practice, University of British Columbia

**SD**, PhD

Associate Professor, Department of Family Practice

University of British Columbia

**JR,** MA, RM

Associate Professor, Midwifery Education Program

Ryerson University

**RL**, MD ChB FRCOG FRCSC FACOG

Professor Emeritus, Department of Obstetrics and Gynaecology

Faculty of Medicine, University of British Columbia

**JK**, PhD

Professor and Research Director

Department of Family and Emergency Medicine

University of Montreal Hospital Research Centre (CRCHUM)

## Abbreviations

PAPHB: Provider attitudes to planned home birth scale
PAPHB-*m*: Provider attitudes to planned home birth scale - multidisciplinary
CNM: Certified nurse-midwife
RM: Registered midwife
OB: Obstetrician
FP: Family physician
MW: Midwife.
